# Monitoring Emerging Risks for Consumers: DIY Projects Using Epoxy Resins in YouTube Videos

**DOI:** 10.3390/ijerph23060723

**Published:** 2026-05-28

**Authors:** Eva Charlotte Rogasch, Marie Bergner

**Affiliations:** German Federal Institute for Risk Assessment, Max-Dohrn-Strasse 8–10, 10589 Berlin, Germany; marie.bergner@bfr.bund.de

**Keywords:** exposure assessment, risk assessment, study design, dermal exposure

## Abstract

**Highlights:**

**Public health relevance—How does this work relate to a public health issue?**

- Contact allergies to epoxy resins are a problem almost exclusively known in patients with occupational exposure. In recent years, it has been observed that consumers also develop epoxy resin allergies.

**Public health significance—Why is this work of significance to public health?**

- Analyzing YouTube videos that show DIY projects using epoxy resins provides insights into how these resins are handled and whether safety precautions are taken.

**Public health implications—What are the key implications or messages for practitioners, policy makers and/or researchers in public health?**

- There are popular videos showing epoxy resin projects without safety measures being taken. These videos can also serve as tutorials for consumers at home. The absence of protective measures while handling epoxy resins may contribute to the development of contact allergies.
- Social media such as YouTube allow consumers to document and share their activities. Analyzing social media content helps to identify and analyze health risks early by making current trends visible.

**Abstract:**

Epoxy resins are associated with health risks, including skin sensitization and allergies. They are used by consumers in DIY applications such as the making of decorative objects and jewelry. To determine whether those uses pose a risk for consumers, a search on the online platform YouTube was conducted. The results revealed about 200 videos with more than 10,000 views, in which DIY applications of epoxy resins are shown, with a growing number of uploads in recent years. The main DIY applications for epoxy resins were making small decorative objects, jewelry, lamps, tabletops, kitchen countertops and floors. In 41% of the videos, no gloves were worn during the handling of epoxy resins. The analysis of content on YouTube shows that the application of epoxy resins by consumers in the DIY sector may present an emerging risk for consumers’ health.

## 1. Introduction

Epoxy resins have many uses, including uses in adhesives, paints, electric isolation, coatings or uses in the wind energy sector [1]. One of the most frequently used resins are diglycidyl ethers of bisphenol A [1]. When the epoxy resin cures, it creates a thermosetting polymer with high heat resistance and high chemical resistance [1]. Under REACH [Regulation [EC] No 1907/2006 [2], the final resin is identified as a polymer that characteristically contains a residual amount of Bisphenol A [BPA] [2] List of Substances of Very High Concern, Bisphenol A]. The U.S. Code of Federal Legislation lists epoxy resin systems containing in any concentration ethylenediamine, diethylenetriamine, and diglycidyl ethers of molecular weight of less than 200 g/mol as “strong sensitizers” in Title 16 § 1500.13 entry [c], seeing that these substances have a significant potential for causing hypersensitivity [3].

From the occupational background, there are studies on the respiratory effects of epoxy resin ingredients, which is why wearing respiratory masks is recommended [4].

The risk of sensitization exists particularly when handling uncured epoxy resin systems [5], which is why wearing gloves is recommended. Skin sensitization is particularly known from occupational settings [6], with a majority of male patients [7]. Cases of sensitization in private settings have also been described [8], where mostly female patients are affected, mainly through using epoxy resin for creative projects [9]. In the Art and Craft Safety Guide by the U. S. Consumer Product Safety Commission, safety measures for using epoxy resins are recommended, including reading the product label, ensuring ventilation and using gloves and goggles. Possible irritation of lungs and the skin is pointed out as well as the possibility of allergies caused by epoxy resins [10].

For creative projects, an epoxy resin is mixed with a hardener, usually in a 1:1 ratio. The resulting mixture is then poured into molds. Bubbles can form during this process, which can be removed with a toothpick or a hot air gun. The resin then needs to dry and can then be removed from the mold.

Consumers are exposed to chemicals when using consumer products [11]. Information on consumer behavior is essential in evaluating consumer exposure and thus in evaluating possible risks for consumers as for example under the REACH Regulation [2]. Data on consumer behavior can be generated by retrospective questionnaire methods as well as protocol methods [12]. However, those methods are associated with relatively high costs and time frames. At the same time, new risks may emerge due to emerging substances such as microplastics or due to changes in consumer behavior [13]. To identify emerging risks, methods that are both quick and associated with low costs can be advantageous. This study analyses social media content to gain information about consumer behavior and to identify emerging risks.

Many people use videos on social media to discover and learn about new hobbies [14]. With two billion users, YouTube is one of the most important websites in the world [15]. In comparison to other topics, the crowd can successfully distinguish expert DIY (Do-It-Yourself) content from unprofessional content [14]. However, it is possible that certain risks are not taken into account by simply learning online. In one example, a child became aware of the use of epoxy resins for the production of decorative objects through social media and subsequently developed severe allergic contact dermatitis [16]. Other examples are described by Temam et al., especially highlighting the risk of developing epoxy resin allergies for women [9]. The products can be easily ordered online and do not always contain warnings about the risk of skin sensitization [9]. Since social media shows increasing interest of consumers in this hobby, skin sensitization from contact with epoxy resins in a non-occupational setting may become a more relevant public health concern. In order to better assess the way in which the handling of epoxy resins is shown on social media, YouTube videos demonstrating how to use epoxy resin were analyzed.

The focus is on the question of whether this continues to be a phenomenon in a professional context, or whether consumers also need to be better informed about epoxy resins.

## 2. Materials and Methods

### 2.1. Youtube Video Analysis

The YouTube platform was searched for videos with the keywords “Epoxy resin deco DIY”, “Epoxidharz Ideen”, “Epoxy resin health risks” and “Epoxidharz Gesundheit”. Since it was observed that using German keywords also yielded videos in other European languages such as Polish and Greek, German keywords were used to get videos from accounts located in Germany or other accounts located in Europe. Videos were sorted by views, as described by [17].

Only videos with at least 10,000 views were included in the analysis to ensure that the material reflects content with demonstrable audience reach and societal relevance, even though this method may create bias by favoring already popular videos. Shorts were not taken into account. Shorts are videos on YouTube that generally last 60 s up to 3 min. The upload year and the views per video were recorded to measure the popularity of the video. A view on YouTube is counted when an individual, who does not need to have an account, watches at least 30 s [15]. Additionally, the number of comments was noted to get insight into what type of content creates more engagement with viewers. Only users with a YouTube account can comment on videos. Views and comments are among the main indicators for evaluating engagement of users with videos on YouTube [18].

For each video, it was recorded whether the performer was wearing or recommending gloves or face masks or any face protection. The “use of gloves” or “use of face masks/face protection” was considered sufficient if they were visibly worn throughout all handling of the epoxy resin. No further distinction was made regarding the material of the protective equipment or whether the use of protective equipment was mentioned or recommended in the audio part of the video. The audio part of the video was evaluated and it was recorded whether there was just music playing or whether the creator is explaining the task with words or subtitles.

Finally, videos gained by the “Epoxy resin deco DIY”/“Epoxidharz Ideen” searches were sorted by topic, i.e., the type of DIY task carried out and the country of the YouTube account was noted. To evaluate the relevance of epoxy resin content on YouTube, a search for projects using modelling clay was conducted at the same time. The search term “modeling clay deco DIY” was used. For all videos showing DIY projects with modelling clay with more than 10,000 views, the year of the upload, as well as the number of views and comments were recorded.

Since YouTube searches can be influenced by browser history or personal preferences, the search was conducted with browser history and cookies cleared and with no account, as described by Sui et al. [15]. The analysis was conducted between 10 February 2025 and 20 February 2025. All data based on YouTube videos was anonymized. After the aforementioned data was recorded for each video, the link to the URL of that video as well as the name of the account of the creator were deleted to ensure that no identification of individual creators is possible from the processed data.

### 2.2. Statistical Analysis

All statistical analyses were performed using R Version 4.5.1 [19], with the significance level set at *p* = 0.05. To examine the relationship between the video’s country of origin or topic of the video and the use of protective gloves, Fisher’s Exact Test was employed, accounting for small sample sizes in some categories. For significant results, post hoc analyses were conducted to identify specific differences between countries. Pairwise Fisher test comparisons were performed using the rstatix package in R, with *p*-values adjusted via the Bonferroni correction to maintain the family-wise error rate.

To analyze differences in viewer engagement (number of views) between videos with and without wearing gloves, the Wilcoxon rank-sum test (Mann–Whitney U test) was employed. This non-parametric approach was selected because the engagement metrics exhibited a strong positive skew, violating the assumption of normality required for a *t*-test. It was also used to analyze differences in number of views and comments between videos gained with the “epoxy resin deco DIY” and “modelling clay deco DIY” searches.

To examine the independent effects of wearing gloves and country of origin on video views and to control for potential confounding factors, a multiple linear regression model was applied. Video views served as the dependent variable, with glove use and country of origin included as predictors. This multivariate approach allowed for determining whether the use of gloves exerted a significant impact on view counts when adjusting for country-specific variations.

To evaluate the relationship between video topics and view or comment counts, a Kruskal–Wallis H-test was performed. This non-parametric method was chosen due to the non-normal distribution of the view data. Significant results were further analyzed using Dunn’s post hoc test with a Bonferroni correction to identify specific differences between individual topics. The Bonferroni correction was used to ensure that significant results were not simply due to the high number of comparisons (random effects). The relationship between video duration and view counts was analyzed using Spearman’s rank correlation coefficient (rho). Results of all statistical tests are found in Appendix A.

## 3. Results

The search revealed 15 YouTube videos with over 10,000 views when searching for “epoxy resin health risks” or “Epoxidharz Gesundheit” (Table 1). In these videos, the creator either talks about their own negative experiences with epoxy resin (N = 3) or gives professional advice about the protective measures that must be taken to ensure safe use of resin (N = 12).

With 188 videos, searching for “epoxy resin deco DIY” and “Epoxidharz Ideen” yielded considerably more results (Table 1). In the majority of these videos (N = 125) music plays in the background and the activities are shown, not described with words. In some cases, there are subtitles describing the activity (N = 55). Some of the videos are compilations that show the making of several epoxy resin objects.

Table 1 shows the results of the keyword search on YouTube, where searches for “epoxy resin deco DIY” and “Epoxidharz Ideen” yielded higher numbers of videos, views, and comments than “epoxy resin health risks” and “Epoxidharz Gesundheit”. However, a comparison of the median and interquartile range (IQR) reveals variability in the number of views and comments for individual videos, independent of the search terms. In most cases, videos discussing risks from the “epoxy resin health risks” or “Epoxidharz Gesundheit” search recommend using gloves and face masks. With 59% of the “epoxy resin deco DIY”/“Epoxidharz Ideen”, the majority of videos demonstrate wearing gloves, but face protection is only worn in 13.3% of the videos (Table 1). In some of the videos, gloves are shown but their use is not explicitly mentioned. This is partly due to the fact that in a number of videos (N = 81) there is only music accompanying the visual demonstration of the project and no verbal or written description of the project as mentioned above. “Epoxy resin health risks” and “Epoxidharz Gesundheit” video durations are slightly shorter than videos from the “epoxy resin deco DIY” and “Epoxidharz Ideen” search (Table 1).

**Figure 1:**
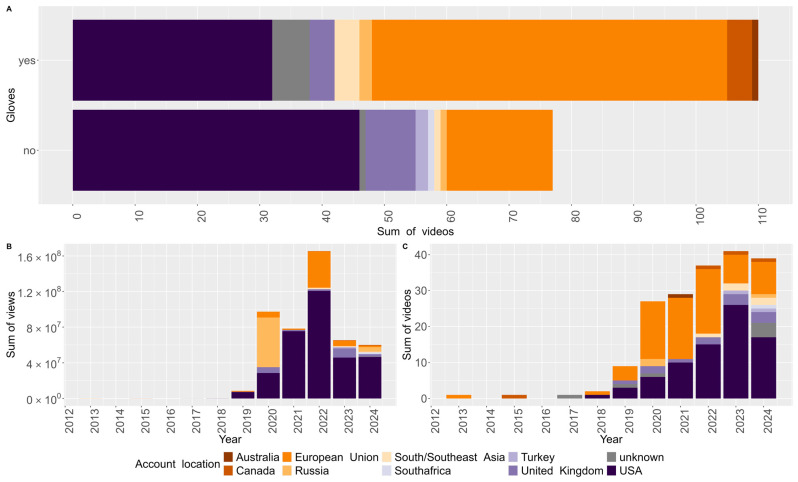
(**A**) Sum of videos in which gloves were worn or not. (**B**) Sum of views of epoxy resin DIY videos during the years 2012–December 2024 and (**C**) Sum of uploaded videos on epoxy resin DIYs during the years 2012–December 2024. Colors refer to the location of the account. In some cases, the countries were summarized as follows: European Union includes videos from Austria, Belgium, Croatia, Cyprus, Germany, Poland and Portugal. South/Southeast Asia includes videos from India and Pakistan, and from the Philippines.

Figure 1 shows the results from “epoxy resin deco DIY” and “Epoxidharz Ideen” search depending on the locations of the video accounts. In general, most videos came from locations in the U.S. and Europe. A Fisher test revealed significant differences in wearing gloves between the countries based on number of videos with gloves or without gloves (*p*-value = 0.0005). Subsequent pairwise post hoc comparisons with Bonferroni correction indicated significant differences specifically between Europe and the U.S. (*p*-value = 0.0005). Gloves were worn significantly more often in Europe compared to the U.S. Looking at the development over the years, beginning in 2012, the number of epoxy resin videos increased, then in 2024 slightly fewer videos were found within the search frame of the study. Looking at the number of views in Figure 1, most views were gained by videos uploaded in 2022, while most videos were uploaded in 2023. Videos from the U.S. got the most views and there was also one video from Russia uploaded in 2020 which gained a remarkably high view count of 55,600,000. A Wilcoxon rank sum test showed that videos without gloves received significantly more views than those with gloves (W = 5627, *p* = 0.0003). The median of views of videos with gloves (N = 76,500) was significantly lower than the median of views of videos without gloves (N = 520,000). To examine the influence of gloves while controlling for country-specific differences, a linear regression model was used. The model showed (F(12,175) = 2.39, *p*-value: 0.007) that taking the country into account, gloves do not have a significant influence on the number of views, so that country-specific reasons lead to higher view counts in videos from the U.S. and not the lack of gloves.

The videos could be divided by topic resulting in eight different categories, namely creating decorative objects such as paperweights, coasters or ornaments (N = 96), making a new floor (N = 5), creating jewelry (N = 23), renovating a kitchen countertop (N = 5), creating a lamp (N = 18), creating a tabletop (N = 33), about technical support (N = 7) and one video of a creator warning about using epoxy resins because of health hazards, which was the only video found also in the “Epoxy resin health risk/Epoxidharz Gesundheit” search.

This video received 11,390 comments in total, showing intensive interaction within the community which indicates a high relevance of the topic for the viewers.

**Figure 2:**
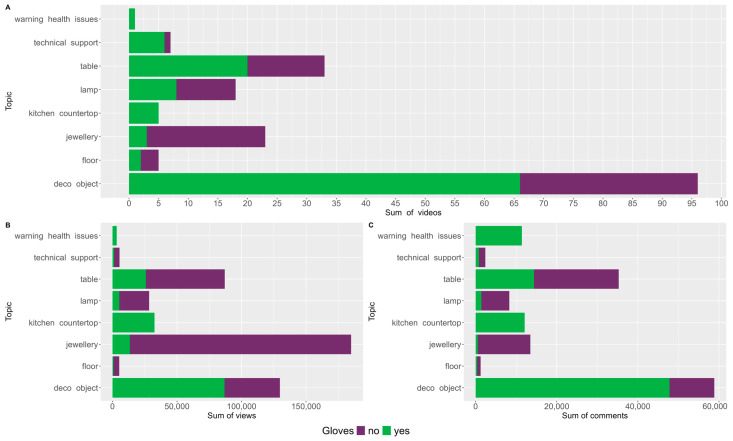
Analysis of YouTube videos found with the keywords “Epoxy resin deco DIY” and “Epoxidharz Ideen”, showing how to use epoxy resins at home and if gloves were worn in those videos (green) or not (violet). (**A**) total number of videos per category found; (**B**) total number of views per category found, for better readability the numbers were divided by 1000; (**C**) total number of comments per category found.

While making decorative objects was the topic in most of the videos, making jewelry had received by far the most views (Figure 2). In all five videos showing the making of kitchen countertops, gloves were worn. In the videos in which a floor was made, gloves were used in two out of five videos. In videos in which a table top was created, gloves were worn in 20 out of 33 videos. Those three categories are the tasks for which a higher product amount is needed in comparison to the smaller tasks of making jewelry, at least when the making of a single object is considered. The number of videos in which gloves are used for these tasks is not significantly higher than the average of 59% of all videos (N = 188) which show gloves being used.

A Fisher test revealed significant differences between the number of videos where gloves were worn depending on the topic of the video (*p*-value = 0.0005). Post hoc tests showed significant differences on the wearing of gloves between videos on deco objects and jewelry, kitchen countertop and jewelry, table and jewelry as well as technical support and jewelry. In all cases, in jewelry videos gloves were worn significantly less often than in the other topics.

A Kruskal–Wallis test was performed to analyze whether specific topics received significantly more views than other. Overall, the test revealed a significant effect of video topic on view counts (H(7) = 26.582, *p*-value = 0.0004). Dunn’s post hoc test subsequently showed that these differences existed primarily between videos on deco objects and kitchen countertops or tables. Deco object videos received significantly more views than the other topics. Similarly, a Kruskal–Wallis test on differences between the number of comments on different topics showed a significant effect (H(7) = 23.362, *p*-value = 0.001). A post hoc test, after Bonferroni correction, showed that there was only a significant difference in the number of comments between “deco object” and “kitchen countertop”, with “deco object” videos receiving significantly more comments.

Spearman’s rank correlation was conducted to assess the relationship between video duration and view counts. The analysis revealed no significant correlation between the two variables (rho = −0.03, *p* = 0.69). These findings suggest that the length of the video did not influence its popularity within the studied sample.

To evaluate the relevance of epoxy resins in the wider field of DIY activities by consumers, a search for modeling clay deco DIY was carried out on YouTube. Modelling clay was chosen since it can be used for similar tasks. Both modelling clay and epoxy resins can be used for making jewelry or decorative objects. Again, only videos with more than 10,000 views were considered. This search yielded a total of 41 videos, i.e., fewer than the videos found for “epoxy resin deco DIY” by a about a factor of five (Table 2).

“Epoxy resin deco DIY” videos achieve significantly higher view counts than “modelling clay deco DIY” videos (W = 2208, *p*-value = 0.04). However, the number of comments per video did not differ significantly between the two keyword searches (W = 2037, *p*-value = 0.2).

**Figure 3:**
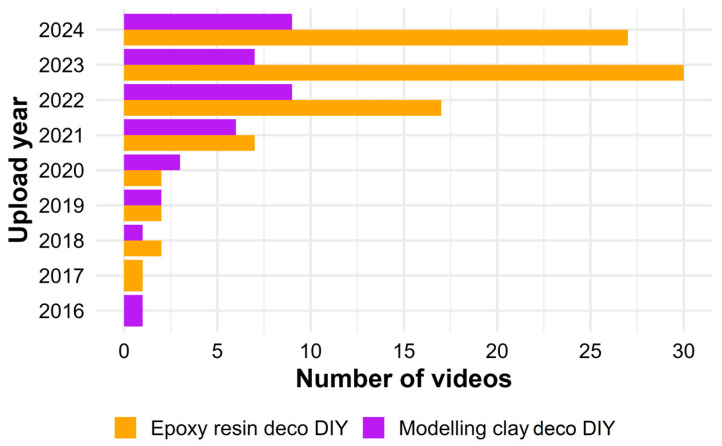
Number of videos uploaded to YouTube each year in the categories of “epoxy resin deco DIY” and “modelling clay DIY”.

A comparison of the number of videos uploaded per year for those two applications showed that the number of videos in which epoxy resins are used steadily increased and by 2021 those videos outnumbered the videos in which modelling clay is used (Figure 3). By 2022, videos on epoxy resin DIY outnumber those on modelling clay by a factor of two and by 2023 epoxy resin videos outnumber videos using modelling clay by a factor of four.

## 4. Discussion

Video evaluation shows that YouTube content with epoxy resin has increased in recent years, even if a slight decline can be observed in 2024. However, since this study was carried out in the beginning of 2025, it is possible that videos posted in 2024 simply had not achieved the threshold of 10,000 views in a relatively short time. The situation may be different if the search on YouTube was carried out again at a later point in time. The increase in YouTube videos since 2020 showing epoxy resin projects can also be related to the corona pandemic, during which the use of YouTube has generally increased significantly [20]. A comparison with the modelling clay search shows that epoxy resin videos have received more attention since 2021 at the latest, indicating the relevance of this type of product for the consumer DIY sector. Studying literature on non-occupational cases of epoxy resin allergy showed that most cases also occurred from 2020 onwards, suggesting a growing prevalence of epoxy resins in DIY applications in this time [8,9,16,21,22,23].

All non-occupational cases of epoxy skin allergies in the literature concern female patients [8,9,16,21,22,23] and related to some kind of casting resin. Indeed, in one case, a girl who later developed allergic contact dermatitis from epoxy resin actually became aware of the product through TikTok videos [16], which at least in this case indicates a clear influence of social media on the consumer.

One non-occupational case of epoxy resin skin sensitization before 2020 was described by Lolatgis and Nixon in 2015 [24]. Here the sensitization was related to a two-component paint resin.

There are popular videos in which safety precautions do not seem to play a role, although YouTube accounts from Europe show wearing gloves significantly more often than accounts located in the U.S.

Face masks or goggles only play a marginal role. These are usually mentioned in videos when health issues are pointed out. Even if it cannot be assumed that all consumers of these videos will actually implement the projects shown, they can serve as both inspiration and role models for epoxy resin beginners. Almost all adults from different age groups can be influenced as is evident from looking at numbers from the U.S., where 81% of adults already use YouTube [20]. Information on hazardous chemicals influences consumer behavior in handling consumer products. Videos in which no gloves are worn can have an effect on how consumers perceive risks associated with epoxy resins and can thus also affect how these products are used [25].

The search “Epoxy resin deco DIY” or “Epoxidharz Ideen” only yielded one video that explicitly warned against the use of epoxy resins in private settings. However, it received a lot of views and also a comparatively large number of comments. This video is an exception, as the targeted search for “Epoxy resin health risk “and “Epoxidharz Gesundheit” only resulted in few videos with significantly lower view and comment numbers. Based on that, the use of epoxy resin as a hobby receives more attention, while only a small, less visible part with fewer views and comments warns about possible risks. A closer look at videos regarding different types of projects showed that jewelry videos significantly less often show the use of gloves and at the same time received the highest number of views.

People who become aware of the epoxy resin hobby via social media possibly do not follow sufficient protective measures due to the lack of professional training. This can be especially true when inspiration is drawn from content online in which no safety measures are proposed. Even if videos from Europe show the wearing of gloves more often, a person searching for epoxy resin videos will be shown videos from all regions of the world and therefore also content in which safety measures are not sufficiently taken into account. A study from Denmark showed that people who were not educated about the safe use of epoxy resins were more likely to not use gloves [26].

The products are easily available on the Internet and the structure of the videos along with the short duration of the videos and the use of music rather than detailed verbalized instructions suggests that the substance is easy to handle. Addressing viewers directly can also encourage consumers to try out the demonstrated project. Consumer-to-consumer reviews can represent a powerful parasocial interaction significantly influencing consumers’ purchasing decisions, as shown in a study from Finland [27]. How products such as epoxy resins are used in videos on YouTube or other social media can directly influence consumer risk perception. Risk perception has been shown to be a key predictor for consumer behavior [28]. A focus group study involving participants from four countries showed that citizens have a general concern regarding chemical exposure on their health and daily life [29]. A second focus group study involving participants from seven countries showed that the perception of risks from exposure to chemicals mostly focuses on exposure via the environment, drinking water and air pollution as well as food. Concerns about chemical exposure from products seemed to play a smaller role [30]. In this context, the way DIY products are used on social media can be of particular significance in shaping risk awareness in consumers.

Consumers will continue to look for inspiration or guidance from social media in the future, both regarding epoxy resins and other DIY or hobby projects. An earlier example of this was the making of homemade slime, which was heavily promoted on social media in 2016 and 2017 and which contained dangerous ingredients such as borates in some cases [31].

Information on the type of project carried out is highly valuable in the estimation of a risk associated with this use, as for example foreseen under the REACH Regulation. To estimate a risk, the evaluation of the associated exposure to a chemical is necessary in addition to the evaluation of a hazard associated with a substance. Various models for estimating exposure are available, all of which require knowledge on how products are used by consumers [32]. ConsExpo is a software tool developed for the assessment of exposure to chemicals from consumer products and is recommended for exposure assessment under REACH [2]. It requires the input of parameters such as exposure duration and the amount of product used, which can be estimated based upon typical scenarios [33]. No default scenarios in ConsExpo exist for most projects typically carried out with epoxy resin. Use by hobbyists is not covered by the scenarios in the ConsExpo DIY Fact sheet. There is a grey area between DIY tasks and hobbies, which differ by frequency and duration of the use. However, parameters may be derived from the typical projects found in both the YouTube analysis and comparison with other default scenarios such as the use of two-component glue.

## 5. Limitations

YouTube constitutes a highly dynamic platform and fully replicable sampling procedures are challenging due to the limited transparency of the platform regarding the recommendation and ranking algorithms, but also due to constant uploads of new content to the platform. The sampling therefore reflects a time-bound snapshot of highly visible content. However, taking only videos with more than 10,000 views into account may underrepresent emerging and niche content. It can be assumed that videos with a high number of views and comments will be among those that are most often found as a result of searching for a given term. It must however be noted that the specific videos a user will be shown most likely also depend on the viewers search history, which will be different for every single user and cannot be replicated in a search like the one presented in this work.

The focus on the topic “epoxy resin” did not take into account that the popularity of individual YouTubers can also influence view or comment counts. However, YouTubers potentially select content that is likely to generate clicks [17], which is why this aspect was not examined in more detail at this point.

## 6. Conclusions

We have shown that the analysis of social media content allows for a valuable view on emerging risks in the consumer sector. The analysis of content on YouTube shows that the use of epoxy resins in DIY applications is becoming increasingly popular. At the same time, influential content on YouTube shows handling of the substance without safety precautions.

Carrying out similar projects at home may present an emerging health risk for consumers. It is therefore important to keep an eye on developments and trends in social media and, if necessary, to initiate further measures for consumer protection.

## Figures and Tables

**Table 1:** Comparison of YouTube video search on different kind of epoxy resin videos.

	Keywords YouTube Search	
	“Epoxy Resin Health Risks” “Epoxidharz Gesundheit”	“Epoxy Resin Deco DIY” “Epoxidharz Ideen”
Videos with more than 10,000 views	15	188
Views in total	4,452,460	476,591,000
Views per video, median (IQR)	49,250 (78,450)	151,500 (1,193,025)
Comment number in total	13,939	142,633
Comments per video, median (IQR)	144 (138)	122 (445.75)
Number of videos in which gloves are recommended/worn	12 (80%)	111 (59%)
Number of videos in which face masks/protection are recommended/worn	11 (73%)	26 (13.8%)
Duration of the videos in minutes in total	154	2876
Duration per video, median (IQR)	9 (8.25)	12 (8.25)

**Table 2:** Comparison of search results generated on YouTube for “epoxy resin deco DIY” and “modelling clay deco DIY”.

	Keywords YouTube Search	
	Epoxy Resin Deco DIY	Modelling Clay Deco DIY
Number of videos	88	41
Median views (IQR)	643,500 (2,353,000)	171,000 (364,700)
Median comments (IQR)	213.5 (740)	115 (404)
Duration per video median (IQR)	13 (6.25)	10 (9)
Maximum views/1 video	68,000,000	14,800,000
Maximum comments/1 video	14,093	1780

## Data Availability

The original contributions presented in this study are included in the article. Further inquiries can be directed to the corresponding author.

## Appendix A. Details on Statistical Tests

**Table A1:** Cross-tabulation of whether gloves were worn in different countries and result of Fisher’s exact test.

Country	Gloves Used in the Video?	
	No	Yes
Australia	0	1
Canada	0	4
Europe	17	58
India	1	2
NA	1	6
Pakistan	0	1
Philippines	0	1
Russia	1	2
South Africa	1	0
Turkey	2	0
United Kingdom	8	4
Fisher’s Exact Test for Count Data with simulated *p*-value (based on 2000 replicates), alternative hypothesis: two.sided	*p*-value = 0.0005	

**Table A2:** Pairwise post hoc comparisons of glove use frequency by origin country of the video (pairwise Fisher test).

Comparison Group 1	Comparison Group 2	Sample Size	*p*-Value	*p*-Value Adjusted	Significance
Australia	Canada	5	1	1	ns
Australia	Europe	76	1	1	ns
Australia	India	4	1	1	ns
Australia	NA	8	1	1	ns
Australia	Pakistan	2	1	1	ns
Australia	Philippines	2	1	1	ns
Australia	Russia	4	1	1	ns
Australia	South Africa	2	1	1	ns
Australia	Turkey	3	0.333	1	ns
Australia	United Kingdom	13	0.385	1	ns
Australia	USA	79	0.418	1	ns
Canada	Europe	79	0.572	1	ns
Canada	India	7	0.429	1	ns
Canada	NA	11	1	1	ns
Canada	Pakistan	5	1	1	ns
Canada	Philippines	5	1	1	ns
Canada	Russia	7	0.429	1	ns
Canada	South Africa	5	0.2	1	ns
Canada	Turkey	6	0.0667	1	ns
Canada	United Kingdom	16	0.0769	1	ns
Canada	USA	82	0.0337	1	ns
Europe	India	78	0.55	1	ns
Europe	NA	82	1	1	ns
Europe	Pakistan	76	1	1	ns
Europe	Philippines	76	1	1	ns
Europe	Russia	78	0.55	1	ns
Europe	South Africa	76	0.237	1	ns
Europe	Turkey	77	0.0584	1	ns
Europe	United Kingdom	87	0.00387	0.255	ns
Europe	USA	153	7.21 × 10^−6^	0.000476	***
India	NA	10	1	1	ns
India	Pakistan	4	1	1	ns
India	Philippines	4	1	1	ns
India	Russia	6	1	1	ns
India	South Africa	4	1	1	ns
India	Turkey	5	0.4	1	ns
India	United Kingdom	15	0.525	1	ns
India	USA	81	0.569	1	ns
NA	Pakistan	8	1	1	ns
NA	Philippines	8	1	1	ns
NA	Russia	10	1	1	ns
NA	South Africa	8	0.25	1	ns
NA	Turkey	9	0.0833	1	ns
NA	United Kingdom	19	0.0573	1	ns
NA	USA	85	0.0416	1	ns
Pakistan	Philippines	2	1	1	ns
Pakistan	Russia	4	1	1	ns
Pakistan	South Africa	2	1	1	ns
Pakistan	Turkey	3	0.333	1	ns
Pakistan	United Kingdom	13	0.385	1	ns
Pakistan	USA	79	0.418	1	ns
Philippines	Russia	4	1	1	ns
Philippines	South Africa	2	1	1	ns
Philippines	Turkey	3	0.333	1	ns
Philippines	United Kingdom	13	0.385	1	ns
Philippines	USA	79	0.418	1	ns
Russia	South Africa	4	1	1	ns
Russia	Turkey	5	0.4	1	ns
Russia	United Kingdom	15	0.525	1	ns
Russia	USA	81	0.569	1	ns
South Africa	Turkey	3	1	1	ns
South Africa	United Kingdom	13	1	1	ns
South Africa	USA	79	1	1	ns
Turkey	United Kingdom	14	1	1	ns
Turkey	USA	80	0.514	1	ns
United Kingdom	USA	90	0.756	1	ns

*p*-values were adjusted using the Bonferroni correction to account for multiple testing. Significance levels: *p* < 0.001 ***, ns = non-significant.

**Table A3:** Gloves used in the video in correlation with view counts and Wilcoxon rank sum test results.

Gloves Used in the Video?	View Counts Median (Total Number of Views)
no	520,000 (307,276,100)
yes	76,500 (169,314,900)
Wilcoxon rank sum test with continuity correction	W = 5627, *p*-value = 0.0002263

**Table A4:** Results of the multiple linear regression model to predict video views based on country of origin of the video and the use of gloves.

Variable	Unstandardized Coefficient	Standard Error	T-Statistic	*p*-Value
Intercept	1,712,742.95	7,521,484.99	0.2277134	0.82
Canada	398,975	8,297,521.52	0.04808364	0.96
Europe	372,287.598	7,475,976.11	0.04979786	0.96
India	50,952.3501	8,579,320.97	0.00593897	1.00
Pakistan	706,800	10,495,626.8	0.06734233	0.95
Philippines	768,800	10,495,626.8	0.07324956	0.94
Russia	1,968,8285.7	8,579,320.97	2.29485361	0.02 *
South Africa	187,257.05	105,66542.8	0.0177217	0.99
Turkey	−868,742.95	9,171,274.82	−0.09472434	0.92
United Kingdom	782,021.367	7,767,422.83	0.10067964	0.92
United States of America	3,143,131.08	7,503,647.06	0.41888045	0.68
No country given in the description of the YouTube canal	36,793.8643	7,935,868.87	0.0046364	1.00
Gloves used in the video?—with Answer: yes	−1,701,542.95	1,222,148.02	−1.39225603	0.17

Notes: N = 188, Overall model: F(12,175) = 2.39, *p* = 0.007 **; R^2^ = 0.141, Adjusted R^2^ = 0.082; The reference category for the intercept consists of videos from Australia with no gloves worn. *p*-values were adjusted using the Bonferroni correction to account for multiple testing. Significance levels: *p* < 0.5 *, *p* < 0.01 **.

**Table A5:** Cross-tabulation of whether gloves were worn in videos with different topics and result of Fisher’s exact test.

Topic of the Video	Gloves Used in the Video?	
	No	Yes
Deco object	30	66
Floor	3	2
Jewelry	20	3
Kitchen countertop	0	5
Lamp	10	8
Table	13	20
Technical support	1	6
Warning health issues	0	1
Fisher’s Exact Test for Count Data with simulated *p*-value (based on 2000 replicates), alternative hypothesis: two-sided	*p*-value = 0.0005	

**Table A6:** Pairwise post hoc comparisons of glove use frequency by topic of the video (pairwise Fisher test).

Comparison Group 1	Comparison Group 2	Sample Size	*p*-Value	*p*-Value Adjusted	Significance
deco object	floor	101	0.327	1	ns
deco object	jewelry	119	1.76 × 10^−6^	4.93 × 10^−5^	****
deco object	kitchen countertop	101	0.318	1	ns
deco object	lamp	114	0.061	1	ns
deco object	table	129	0.4	1	ns
deco object	technical support	103	0.672	1	ns
deco object	warning health issues	97	1	1	ns
floor	jewelry	28	0.207	1	ns
floor	kitchen countertop	10	0.167	1	ns
floor	lamp	23	1	1	ns
floor	table	38	0.632	1	ns
floor	technical support	12	0.222	1	ns
floor	warning health issues	6	1	1	ns
jewelry	kitchen countertop	28	0.00057	0.016	*
jewelry	lamp	41	0.0357	1	ns
jewelry	table	56	0.000388	0.0109	*
jewelry	technical support	30	0.000884	0.0248	*
jewelry	warning health issues	24	0.167	1	ns
kitchen countertop	lamp	23	0.0457	1	ns
kitchen countertop	table	38	0.144	1	ns
kitchen countertop	technical support	12	1	1	ns
kitchen countertop	warning health issues	6	1	1	ns
lamp	table	51	0.378	1	ns
lamp	technical support	25	0.09	1	ns
lamp	warning health issues	19	0.474	1	ns
table	technical support	40	0.387	1	ns
table	warning health issues	34	1	1	ns
technical support	warning health issues	8	1	1	ns

*p*-values were adjusted using the Bonferroni correction to account for multiple testing. Significance levels: *p* < 0.5 *, *p* < 0.0001 ****, ns = non-significant.

**Table A7:** Kruskal–Wallis Test and Dunn’s Post-hoc comparisons for video views across different video topics.

Dependent Variable	Comparison Group 1	Comparison Group 2	Sample Size Group 1	Sample Size Group 2	Z-Statistic	*p*-Value	*p*-Value Adujsted	Significance
Views	deco object	floor	96	5	0.22014062	0.82576164	1	ns
Views	deco object	jewelry	96	23	2.64462086	0.00817825	0.22899098	ns
Views	deco object	kitchen countertop	96	5	3.34910053	0.00081074	0.02270082	*
Views	deco object	lamp	96	18	1.49024835	0.13615895	1	ns
Views	deco object	table	96	33	3.5982652	0.00032035	0.00896971	**
Views	deco object	technical support	96	7	0.22440313	0.82244363	1	ns
Views	deco object	warning health issues	96	1	1.48079819	0.13866036	1	ns
Views	floor	jewelry	5	23	1.03959966	0.29852594	1	ns
Views	floor	kitchen countertop	5	5	2.26939488	0.02324432	0.65084106	ns
Views	floor	lamp	5	18	0.55742051	0.57724015	1	ns
Views	floor	table	5	33	1.30260163	0.1927108	1	ns
Views	floor	technical support	5	7	−0.02241839	0.98211421	1	ns
Views	floor	warning health issues	5	1	1.26661714	0.20529222	1	ns
Views	jewelry	kitchen countertop	23	5	1.86917428	0.06159857	1	ns
Views	jewelry	lamp	23	18	−0.73462873	0.46256564	1	ns
Views	jewelry	table	23	33	0.41285334	0.67971407	1	ns
Views	jewelry	technical support	23	7	−1.21877118	0.22293105	1	ns
Views	jewelry	warning health issues	23	1	0.85612121	0.39193074	1	ns
Views	kitchen countertop	lamp	5	18	−2.28178875	0.02250182	0.63005083	ns
Views	kitchen countertop	table	5	33	−1.68821812	0.09136937	1	ns
Views	kitchen countertop	technical support	5	7	−2.47364501	0.01337425	0.37447904	ns
Views	kitchen countertop	warning health issues	5	1	−0.0436186	0.96520842	1	ns
Views	lamp	table	18	33	1.17169967	0.24131766	1	ns
Views	lamp	technical support	18	7	−0.66208676	0.50791562	1	ns
Views	lamp	warning health issues	18	1	1.07622837	0.28182514	1	ns
Views	table	technical support	33	7	−1.53377895	0.12508404	1	ns
Views	table	warning health issues	33	1	0.75109704	0.45259426	1	ns
Views	technical support	warning health issues	7	1	1.3101754	0.1901365	1	ns

Global Kruskal–Wallis test: data: Views by Topic; Kruskal–Wallis chi-squared = 26.582, df = 7, *p*-value = 0.0003961; *p*-values were adjusted using the Bonferroni correction to account for multiple testing. Significance levels: *p* < 0.5 *, *p* < 0.01 **, ns = non-significant.

**Table A8:** Kruskal–Wallis Test and Dunn’s Post hoc comparisons for video comment numbers across different video topics.

Dependent Variable	Comparison Group 1	Comparison Group 2	Sample Size Group 1	Sample Size Group 2	Z-Statistic	*p*-Value	*p*-Value Adujsted	Significance
Comment number	deco object	floor	96	5	0.096862925	0.922835252	1	ns
Comment number	deco object	jewelry	96	23	0.489800371	0.624275168	1	ns
Comment number	deco object	kitchen countertop	96	5	3.33803577	0.000843729	0.023624408	*
Comment number	deco object	lamp	96	18	1.900935042	0.057310522	1	ns
Comment number	deco object	table	96	33	3.021937317	0.002511626	0.070325515	ns
Comment number	deco object	technical support	96	7	−0.172853408	0.862766655	1	ns
Comment number	deco object	warning health issues	96	1	1.888940387	0.058899817	1	ns
Comment number	floor	jewelry	5	23	0.140396055	0.888347075	1	ns
Comment number	floor	kitchen countertop	5	5	2.3507815	0.018734031	0.524552873	ns
Comment number	floor	lamp	5	18	0.87794682	0.379972579	1	ns
Comment number	floor	table	5	33	1.178098441	0.238757363	1	ns
Comment number	floor	technical support	5	7	−0.191455114	0.848169043	1	ns
Comment number	floor	warning health issues	5	1	1.692755687	0.090501981	1	ns
Comment number	jewelry	kitchen countertop	23	5	2.872694364	0.004069876	0.11395654	ns
Comment number	jewelry	lamp	23	18	1.190187611	0.233972662	1	ns
Comment number	jewelry	table	23	33	1.826370993	0.067794398	1	ns
Comment number	jewelry	technical support	23	7	−0.420188411	0.67434782	1	ns
Comment number	jewelry	warning health issues	23	1	1.747460502	0.080557491	1	ns
Comment number	kitchen countertop	lamp	5	18	−2.063084142	0.039104635	1	ns
Comment number	kitchen countertop	table	5	33	−1.919980172	0.054860404	1	ns
Comment number	kitchen countertop	technical support	5	7	−2.730589338	0.00632212	0.177019347	ns
Comment number	kitchen countertop	warning health issues	5	1	0.335531355	0.737224296	1	ns
Comment number	lamp	table	18	33	0.414803283	0.678285916	1	ns
Comment number	lamp	technical support	18	7	−1.2480569	0.212010221	1	ns
Comment number	lamp	warning health issues	18	1	1.372876827	0.169790642	1	ns
Comment number	table	technical support	33	7	−1.628050627	0.103514151	1	ns
Comment number	table	warning health issues	33	1	1.269856057	0.204135908	1	ns
Comment number	technical support	warning health issues	7	1	1.839422703	0.065853037	1	ns

Global Kruskal–Wallis test: data: Comments by Topic; Kruskal–Wallis chi-squared = 23.362, df = 7, *p*-value = 0.001474; *p*-values were adjusted using the Bonferroni correction to account for multiple testing. Significance levels: *p* < 0.5 *, ns = non-significant.

**Table A9:** Spearman’s rank correlation coefficient (*rho*) to show if video duration and video views correlate significantly.

Variable 1	Variable 2	*rho*	*p*	Significance
Video Duration	Video Views	−0.03	0.686	ns

Significance levels: ns = non-significant.

**Table A10:** Comparison of “Epoxy resin deco DIY” video search and “Modelling clay deco DIY” in correlation with view and comment counts and Wilcoxon rank sum test results.

Keyword Search	View Counts Median (Total Number of Views)	Comment Counts Median (Total Number of Comments)
Epoxy resin deco DIY	643,500 (322,597,530)	213.5 (76,598)
Modelling clay deco DIY	171,000 (41,551,600)	115 (11,588)
Wilcoxon rank sum test with continuity correction	W = 2208, *p*-value = 0.04124	W = 2037, *p*-value = 0.2396
